# Evidence for a Cross-Talk Between Cytosolic 5′-Nucleotidases and AMP-Activated Protein Kinase

**DOI:** 10.3389/fphar.2020.609849

**Published:** 2020-12-21

**Authors:** Marcella Camici, Mercedes Garcia-Gil, Simone Allegrini, Rossana Pesi, Maria Grazia Tozzi

**Affiliations:** ^1^ Unità di Biochimica, Dipartimento di Biologia, Università di Pisa, Pisa, Italy; ^2^ Unità di Fisiologia Generale, Dipartimento di Biologia, Università di Pisa, Pisa, Italy

**Keywords:** cytosolic 5′-nucleotidases I and II, AMP-activated kinase, purine cycle, body weight, muscle contraction

## Introduction

**FIGURE 1:**
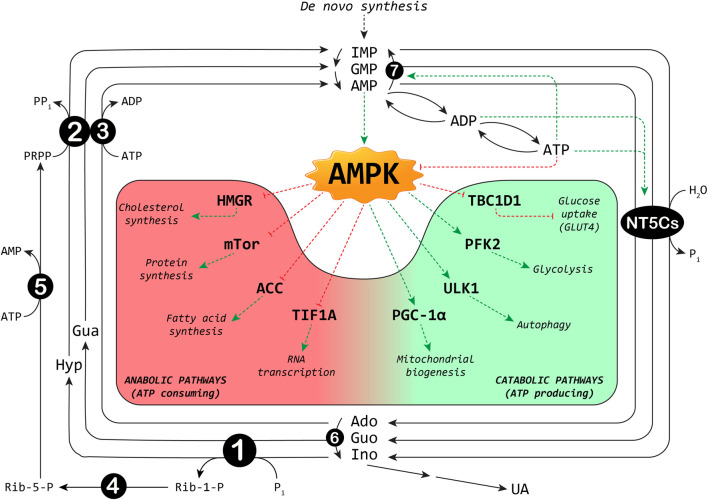
Interplay between purine cycles and AMPK. A selection of metabolic pathways regulated by AMPK is shown: the red background includes the anabolic pathways switched off, and the green background the catabolic pathways switched on by AMPK. ACC: acetyl-CoA carboxylase; Ado: adenosine; ADP: adenosine-5′-diphosphate; AMP: adenosine-5′-monophosphate; ATP: adenosine-5′-triphosphate; Gua: guanine; Guo: guanosine; GMP: guanosine-5′-monophosphate; HMGR: 3-hydroxy-3-methylglutaryl-Coenzyme A reductase; Hyp: hypoxanthine; IMP: inosine-5′-monophosphate; Ino: inosine; NT5Cs: cytosolic 5′-nucleotidase I and II; mTOR: mammalian target of rapamycin; PFK2: 6-phosphofructo-2-kinase; Pi: inorganic phosphate; PGC-1α: peroxisome proliferator-activated receptor-gamma coactivator-*1alpha*; PPi: inorganic pyrophosphate; PRPP: phosphoribosylpyrophosphate; Rib-1-P: ribose-1-phosphate; Rib-5-P: ribose-5-phosphate; TBC1D1: TBC1 domain family member one; TIF1A: transcription intermediary factor-1α; UA: uric acid; ULK1: Unc-51 like autophagy activating kinase 1. Enzymes involved are indicated by numbers inside circles: 1) Purine nucleoside phosphorylase; 2) Hypoxanthine-guanine phosphoribosyltransferase; 3) Adenosine kinase; 4) Phosphoribomutase; 5) PRPP synthetase; 6) Adenosine deaminase; 7) AMP deaminase. Dotted red lines: inhibition; dotted green lines: activation.

The maintenance of the correct balance of nucleotide pools is essential for many vital functions (Bester et al., 2011; Garcia-Gil et al., 2018; Camici et al., 2019). The control of several enzyme activities required for nucleotide metabolism contributes to this homeostasis. Among the involved enzymes, cytosolic 5′-nucleotidases (NT5Cs) play a central role in the regulation of the purine nucleotide pool (Figure 1). The major NT5Cs acting on purine nucleotides are cytosolic 5′-nucleotidase I (NT5C1), which exerts its action mainly in skeletal muscle, and cytosolic 5′-nucleotidase II (NT5C2), which is ubiquitously expressed. The preferred substrate for NT5C1 is AMP, with a K_M_ in the millimolar range (Hunsucker et al., 2001; Tkacz-Stachowska et al., 2005). Although preferring IMP and GMP as substrates (K_M_ in the micromolar range) (Tozzi et al., 2013), NT5C2 catalyses also the hydrolysis of the phosphoester bond of AMP (with a K_M_ in the millimolar range) (Tozzi et al., 2013). The rate of the IMP-GMP cycle (Figure 1) which regulates the intracellular purine nucleotide concentrations, depends on NT5C2 activity (Barsotti et al., 2003). In fact, in the presence of high energy charge, NT5C2 catalyses the catabolism of excess IMP, synthesized by *de novo* or salvage pathways, while allowing for IMP and AMP accumulation in case of low energy charge (Pesi et al., 1994; Allegrini et al., 2004; Wallden and Nordlund, 2011; Camici et al., 2018). For the regulation of the AMP cycle, both NT5C1 and NT5C2 activities are involved (Figure 1). In the last decades growing evidence indicates the central “energy sensing” role played by the AMP-activated protein kinase (AMPK) (Hardie et al., 2012; Garcia and Shaw, 2017). AMPK is a heterotrimer composed of the catalytic α (α1 or α2), the regulatory β (β1 or β2) and the γ subunits (γ1, γ2 or γ3). Alterations in the AMP:ATP ratio are perceived by the γ subunit of AMPK which contains three AMP binding sites, two of which exchangeable with ATP (Xiao et al., 2007). The binding of AMP further increases the kinase activity of AMPK both allosterically and inhibiting its dephosphorylation (Sanders et al., 2007). The major upstream kinases that activate AMPK by phosphorylation of Thr172 (Hawley et al., 1996), are the tumour suppressor kinase LKB1 (Woods et al., 2003) and the Ca^2+^/calmodulin-dependent kinase kinase β (Hawley et al., 2005). AMPK is activated when the cellular energy charge is low and, acting on several protein targets, this protein kinase switches off the anabolic pathways that require ATP and switches on the catabolic pathways that produce ATP (Figure 1). AMPK activation brings about an increase in muscular glucose uptake and fatty acid oxidation, making AMPK activators useful tools for the treatment of type 2 diabetes (Coughlan et al., 2014). In addition, AMPK activation may be responsible for some of the tumour suppression functions of LKB1 (Hardie and Alessi, 2013). Since NT5Cs are the major responsible for the regulation of the AMP level (Kulkarni et al., 2011), it is conceivable that alterations in their activities may affect the numerous signaling pathways triggered by AMPK activation, and thus the regulation of biological processes including muscle contraction, functioning of the nervous system, and control of body weight.

### In Muscle

Gene silencing of *NT5C1A* by shRNA injection and electroporation in mouse tibialis anterior muscle decreased NT5C1A protein expression, increased phosphorylation of AMPK and of its substrate acetyl-CoA carboxylase (ACC), as well as glucose uptake (Kulkarni et al., 2011). Similar results were obtained by using *NT5C2* siRNA in cultured human myotubes. The downregulation of *NT5C2* led to an increase in the AMP:ATP ratio, an increase in AMPK phosphorylation (Thr172), and an increase in ACC phosphorylation (Kulkarni et al., 2011). Overexpression of NT5C1A in human embryonic kidney (HEK293T) cells caused a reduction in the oligomycin-induced increase in AMP and ADP concentrations and a decrease in AMPK activation (Plaideau et al., 2012). Surprisingly, *NT5C1A* and *NT5C2* deletion were not able to potentiate AMPK activation following electrical stimulation in soleus and extensor digitorum longus (EDL) mouse muscles (Kviklyte et al., 2017). AMP:ATP or ADP:ATP ratios in the knockout resting muscles were similar to those of wild type (WT) mice, and contraction did not induce a potentiation of these ratios in the muscle of the knockout animals (Kviklyte et al., 2017). In fact, electrical stimulation induced a 4-fold increase of AMPK activity compared to the resting state both in WT and nucleotidase-deleted muscles. Also, downstream ACC phosphorylation and glucose uptake appeared to increase to the same extent in EDL from electrically stimulated WT and *NT5C2*-or *NTC1A*-knockout mice (Kviklyte et al., 2017). In addition, the effects of the combination of *NT5C1* deletion plus inhibition of AMP deaminase on AMP:ATP ratio and AMPK activity, measured in resting and electrically-stimulated EDL muscle were not different between muscle from WT and knock-out animals (Kviklyte et al., 2017). The authors hypothesized that, during contraction, fluxes through nucleotidases might be too reduced to influence AMP levels and concluded that pharmacological inhibition of AMP-metabolizing enzymes might not be useful for promoting AMPK activation and glucose uptake in muscle of type-2 diabetic patients.

### In Nervous System

In human neural progenitor cells (hNPCs), *NT5C2* knockdown by siRNA increased AMPK protein expression and phosphorylation, and surprisingly, phosphorylation of 40S ribosomal protein S6 (RPS6), without modification in RPS6 expression. It also altered transcription of several genes involved in protein translation (Duarte et al., 2019). RPS6 correlates with mammalian target of rapamycin complex 1 (mTORC1) activation and it is frequently used to estimate the rate of protein translation (Biever et al., 2015). HEK293T cells overexpressing *NT5C2* were used to further investigate the association between *NT5C2* and the regulation of AMPK and RPS6. Duarte et al. (2019) found a decrease in phosphorylated AMPK but not in total AMPK in these cells, and a decrease in total RPS6 protein associated with 300% increase in RPS6 phosphorylation. Therefore, the effect of NT5C2 on RPS6 in HEK293T cells was opposite to that observed in hNPCs. The authors suggested that the increase in RPS6 phosphorylation observed in hNPCs as a consequence of *NT5C2* knockdown could be ascribed to a negative feedback loop leading to increased protein synthesis after an initial arrest in protein synthesis, already described during recovery in muscle (Dreyer et al., 2006). Indeed, endurance exercise in humans increased AMPKα2 activity and immediately decreased protein synthesis. This was followed by increased p70S6K phosphorylation, and increased protein synthesis during the recovery period, 2 h after a bout of exercise (Dreyer et al., 2006). It is worthy to note that since protein synthesis has not been directly measured in hNPCs, it is impossible to know whether the increase in phospho-RPS6 observed after *NT5C2* knockdown (Duarte et al., 2019) does reflect a raise in protein synthesis. Conversely, protein synthesis was dramatically lower in *NT5C2-* knockdown human lung carcinoma (A549) cells compared to control cells (Pesi et al., 2018), but no modification of AMPK activity has been found in these cells, probably as a consequence of an inactivating mutation of LKB1 in A459 cells (Zhong et al., 2006).

*NT5C2* is associated with disorders characterized by psychiatric and psychomotor disturbances such as hereditary spastic paraplegias (HSP) (Garcia-Gil et al., 2018), schizophrenia (Cross-Disorder Group of the Psychiatric Genomics, 2013; Duarte et al., 2016; Duarte et al., 2019) and Parkinson disease. The aberrantly spliced *NT5C2* described by Elsaid et al. (2017) in individuals affected by HSP showed substantial reduction in expression level in the *in vitro* study, indicating marked instability of the mutant NT5C2 protein. The authors suggest that homozygous alteration in *NT5C2* might be necessary to produce central white matter developmental defects (Elsaid et al., 2017). It is interesting to note that knockdown of the NT5C2 homologue in *Drosophila melanogaster* was associated with abnormal climbing behavior when driven by a neuronal promoter, supporting a role for NT5C2 in motility (Duarte et al., 2019). The mechanisms underlying the pathological effects of *NT5C2* mutations are unknown. It could be interesting to obtain information not only on the levels of expression and/or activity of NT5C2, but also on the possible variations of AMP:ATP ratio which could lead to an upregulation of AMPK. A permanent activation of AMPK could result in abnormal development of the nervous system. Indeed, AMPK activation induces apoptosis in hippocampal and neuroblastoma cells (Pesi et al., 2000; Garcia-Gil et al., 2003), and reduces axonal growth (Williams et al., 2011). Moreover, AMPK hyper-activation in differentiated primary neurons reduces the number of synapses and leads to a loss of neuronal network functionality (Domise et al., 2019) and AMPK signaling has been associated with amyotrophic lateral sclerosis, neurodegenerative and psychiatric disorders (Perera and Turner, 2016; Rosso et al., 2016).

### In Body Weight

Body weight of *NT5C1A* ^*−/−*^ and *NT5C2* ^*−/−*^ mice, fed a normal-chow diet, was similar to their WT littermates (Kviklyte et al., 2017). However, *NT5C2* ^*−/−*^ mice fed a high fat diet (HFD) increased their body weight significantly less as compared to WT mice (Johanns et al., 2019). The difference was not due to changes in food consumption or water intake. Although not significant, the authors reported a tendency toward increased AMPK activity in fat pads from *NT5C2* ^*−/−*^ compared with WT mice, both in basal and noradrenaline-stimulated conditions, while a significant increase in AMP concentration was only seen in fat pads from *NT5C2* ^*−/−*^ mice in response to noradrenaline treatment. Consistent with an activation of AMPK, a significant increase in ACC phosphorylation was associated to *NT5C2* deletion and HFD (Johanns et al., 2019).

Genome-wide association studies performed on Japanese subjects revealed that the T-allele of rs11191548 in the *NT5C2* gene was associated with reduced visceral fat area, subcutaneous fat area and total fat area in women (Hotta et al., 2012). Unfortunately, the authors did not measure the activity of NT5C2, therefore we do not know whether the reported single-nucleotide polymorphism affects the function of the enzyme and the level of AMP. Although not supported by the experimental data, it is conceivable to hypothesize an involvement of AMPK, which has been reported to integrate nutrient and hormonal signals to regulate food intake and body weight, both in the hypothalamus and peripheral tissues (Xue and Kahn, 2006).

## Concluding Remarks

The activity of NT5C2 is allosterically regulated by ATP that, at high physiological level, stabilises a very active enzyme conformation (Tozzi et al., 2013). In our opinion, high energy charge activates NT5C2 and AMP deaminase activities, leading to the hydrolysis of the excess of newly synthesized or salvaged nucleotides. At low energy charge, the low activity of both enzymes causes an accumulation of nucleoside monophosphates, particularly AMP, that can either activate AMPK, or be hydrolyzed by NT5C1, releasing adenosine, thus starting the purinergic signaling. In fact, extracellular adenosine, binding to widely distributed receptors (A1, A2A, A2B, and A3) acts not only on metabolic regulation through modulation of cyclicAMP intracellular concentration, but also on fine-tuning of synapses and on the coordination of neuronal networks (Agostinho et al., 2020). An increase of extracellular concentration of adenosine might reflect on many biological processes such as proliferation, regulation of blood flow, inflammation and immunosuppression (Vijayan et al., 2017; Jacobson et al., 2019). Conversely, in some cells or organs, the low NT5C2 activity obtained by silencing, was unable to produce significant AMP accumulation, casting some doubt on the mechanism linking low NT5C2 activity and its metabolic consequences. It will be very interesting to further investigate on these molecular mechanisms, since the knowledge of this matter will support the application of NT5C2 inhibitors not only in cancer but also in pathologies such as metabolic syndrome, obesity and diabetes.

## Author Contributions

MC, MG-G, and MT conceived the opinion and wrote the draft manuscript. SA and RP collected references and drew the figure. All authors discussed the content.

## Funding

This work was supported by local grants of the University of Pisa (ex60%2020).

## Conflict of Interest

The authors declare that the research was conducted in the absence of any commercial or financial relationships that could be construed as a potential conflict of interest.
